# Изменение овариального резерва в процессе комбинированного лечения дифференцированного рака щитовидной железы

**DOI:** 10.14341/probl13592

**Published:** 2025-09-14

**Authors:** М. О. Корчагина, Е. Н. Андреева, А. Р. Елфимова, М. С. Шеремета, Г. А. Мельниченко

**Affiliations:** Национальный медицинский исследовательский центр эндокринологии им. академика И.И. Дедова; I.I. Dedov Endocrinology Research Centre; Национальный медицинский исследовательский центр эндокринологии им. академика И.И. Дедова; Российский университет медицины; I.I. Dedov Endocrinology Research Centre; Russian University of Medicine

**Keywords:** дифференцированный рак щитовидной железы, терапия радиоактивным йодом, осложнения, функция яичников, овариальный резерв, антимюллеров гормон, differentiated thyroid cancer, radioactive iodine therapy, complications, ovarian function, ovarian reserve, anti-Müllerian hormone

## Abstract

**BACKGROUND:**

BACKGROUND: Combined treatment of differentiated thyroid cancer (DTC) may have an impact on the reproductive health of patients, in particular on the ovarian reserve (OR) of childbearing-age women. However, knowledge in this area is still insufficient to create general recommendations and an algorithm for managing this cohort of patients based on their current reproductive status and desire to realize their reproductive potential.

**AIM:**

AIM: To assess ovarian function and OR using anti-Müllerian hormone (AMH) and follicle-stimulating hormone (FSH), luteinizing hormone (LH), estradiol (E2) in dynamics in the early follicular phase in women of reproductive age receiving combined treatment for DTC.

**MATERIALS AND METHODS:**

MATERIALS AND METHODS: In a single-center prospective non-comparative study, the clinical and morphological, anamnestic and laboratory parameters of patients receiving combined treatment for DTC were analyzed. The levels of AMH, FSH, LH and E2 were determined in dynamics – after surgical treatment but no later than one month before radioiodine therapy (RAIT), as well as 3 and 6 months after RAIT on the background of suppressive therapy.

**RESULTS:**

RESULTS: A total of 39 women aged 18 to 40 years with a median age of 32 years [27; 37] undergoing combined treatment for DTC were enrolled in the study. The frequency of transient menstrual cycle disturbances after surgery was 18%, and after RAIT — 38%. According to the post-operative DTC status the majority of patients belonged to ATA intermediate-risk group (69%). In addition, 72% of patients received thyroid hormone withdrawal for a period of 4 weeks as a preparation for RAIT. The average activity of 131I was 3720 MBq [3050; 3838]. The levels of FSH and LH did not differ significantly before and after RAIT (R=NS). The level of E2 decreased significantly 3 months after RAIT (P<0.010), further increasing in 6 months to almost the initial values (P=NS). The level of AMH decreased significantly 3 and 6 months after RAIT compared with baseline values (P<0.001). The median AMH before the treatment was 4.10 ng/ml [2.34; 5.82], the nadir of AMH was observed after 3 months — 2.09 ng/ml [1.05; 3.05], and after 6 months AMH increased to 2.31 ng/ml [1.42; 3.37]. In 29% of patients, the AMH level decreased below the reference after 3 months. The predictor of AMH level below 1.2 ng/ml (reflecting reduced OR) 3 months after RAIT was the patient’s age before RAIT. Using the Juden index, a cut-off point of 31 years was determined.

**CONCLUSION:**

CONCLUSION: The level of AMH decreases significantly after RAIT for DTC, which indicates the effect of the therapy on OR, while age at the time of RAIT is the main predictor of AMH level below 1.2 ng/ml after 3 months.

## ОБОСНОВАНИЕ

Дифференцированный рак щитовидной железы (ДРЩЖ) остается наиболее распространенным вариантом рака щитовидной железы, преобладающим среди женского населения во всех возрастных группах [1]. В настоящее время благодаря улучшению диагностических методик растет выявляемость ДРЩЖ, при этом новые случаи, как правило, представлены папиллярной карциномой [2]. С развитием персонализированного подхода к лечению пациентов с ДРЩЖ прогнозирование исходов проводимой терапии и рисков вторичных осложнений, так же как и профилактика нежелательных явлений и отказ от радионуклидного лечения тогда, когда оно не приносит клинической пользы, — одни из приоритетных задач, которые должны своевременно решаться мультидисциплинарной командой специалистов [3][4].

С учетом откладывания беременности и увеличения числа бесплодных пар в общей популяции остро стоит вопрос об изменении овариального резерва (ОР) у пациенток детородного возраста на фоне комбинированного лечения ДРЩЖ, включающего хирургическое лечение, терапию радиоактивным йодом (РЙТ) и дальнейшую супрессию тиреоидными гормонами, направленную на подавление ТТГ-зависимого роста опухоли [5]. Прогнозирование ятрогенного повреждения яичников, сопровождающегося нарушением фолликулогенеза, стероидогенеза и ускорением процессов атрезии фолликулов со снижением функционального ОР, позволяет рекомендовать меры по сохранению фертильности в случаях, когда это необходимо. Одним из чувствительных и удобных прогностических маркеров, применяемых в настоящее время для оценки ОР, является антимюллеров гормон (АМГ). В отличие от фолликулостимулирующего гормона (ФСГ) и эстрадиола (Е2), которые сильно варьируют в зависимости от дня цикла и отражают поздние изменения в яичниках, служа косвенными показателями снижения числа фолликулов, АМГ секретируется более малыми — антральными и преантральными — фолликулами, и его уровень достаточно стабилен на протяжении всего менструального цикла [6][7].

Снижение ОР, включая пул нерастущих и растущих фолликулов, находится в прямой зависимости от возраста и в норме происходит по мере приближения женщины к менопаузе. Однако ряд эндогенных и экзогенных факторов, а также различные заболевания и их лечение могут способствовать нарушению фолликулогенеза и приводить к более раннему истощению пула фолликулов, что отражается снижением репродуктивного потенциала и сокращением детородного периода [8].

АМГ служит предиктором ответа яичников на овариальную стимуляцию, может использоваться как один из диагностических критериев синдрома поликистозных яичников у взрослых пациенток, а также применяется в качестве маркера снижения ОР в процессе гонадотоксичных методов лечения, в особенности химиотерапии, вследствие которой происходит усиление процессов апоптоза [9–11].

К настоящему времени все еще мало исследований в области изменения ОР и фертильности у пациенток с ДРЩЖ, проходящих комбинированное лечение. Кроме того, из доступной литературы известно, что на территории РФ таких исследований не проводилось. По данным зарубежного систематического обзора и метаанализа 2021 г., включающего 4 исследования в области оценки ОР у женщин с ДРЩЖ, прошедших адъювантную РЙТ, выявлено значимое снижение АМГ к 3 месяцу после радионуклидного лечения при сравнении с исходными показателями (Р<0,0001). В последующем АМГ также оставался сниженным к 6 и 12 мес. после терапии (Р=0,003 и Р<0,0001 соответственно) [12].

При этом, несмотря на выявляемые изменения, все еще не существует четких рекомендаций относительно внедрения мер по сохранению фертильности в когорте пациенток с ДРЩЖ, равно как не существует доказательной базы о том, что такие меры не требуются. При этом хорошо известно, что хоть уровень АМГ и не позволяет судить о шансах наступления беременности естественным путем, его низкие значения (<1,2 нг/мл) отражают сниженное число фолликулов в яичниках и служат предиктором бедного ответа яичников на процедуру стимуляции овуляции в рамках программ вспомогательных репродуктивных технологий, а также коррелируют с кумулятивным коэффициентом живорождения у женщин со сниженным овариальным резервом независимо от их возраста [13][14].

## ЦЕЛЬ ИССЛЕДОВАНИЯ

Целью настоящего исследования стала оценка ОР и функционального состояния яичников с помощью динамического определения уровня АМГ, ФСГ, ЛГ и Е2 у женщин репродуктивного возраста, получающих комбинированную терапию по поводу ДРЩЖ.

## МАТЕРИАЛЫ И МЕТОДЫ

## Место и время проведения исследования

Место проведения. Исследование проведено в ГНЦ РФ ФГБУ «НМИЦ эндокринологии» Минздрава РФ в отделении радионуклидной терапии (РНТ).

Время исследования. Исследование проводили с 2023 по 2024 гг.

## Изучаемые популяции (одна или несколько)

Целевая популяция определялась критериями включения и невключения.

Критерии включения: женщины; возраст — от 18 до 40 лет включительно; ДРЩЖ (МКБ-10–С73), установка диагноза произведена в ходе планового патологоанатомического исследования операционного материала; проведение комбинированного лечения ДРЩЖ, включающего оперативное лечение, РЙТ и супрессивную терапию.

Критерии невключения: только хирургическое лечение ДРЩЖ, установленный диагноз бесплодия, операции на яичниках или лучевая терапия на органах малого таза в анамнезе, синдром поликистозных яичников, беременность, лактация, прием комбинированных оральных контрацептивов (КОК) на момент обследования или завершение приема КОК позднее чем за 2 мес. до начала обследования, прием заместительной гормональной терапии (ЗГТ) половыми стероидами или завершение приема ЗГТ позднее чем за 2 мес. до начала обследования.

Способ формирования выборки — сплошной.

Дизайн исследования — одноцентровое проспективное несравнительное исследование. Дизайн исследования представлен на рисунке 1.

**Figure:**
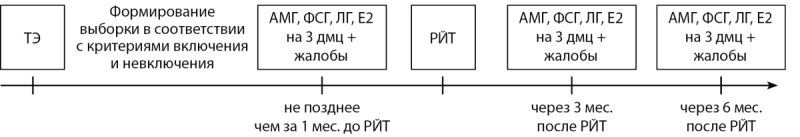
Рисунок 1. Дизайн исследования. ТЭ — тиреоидэктомия; дмц — день менструального цикла.

Из 39 пациенток, включенных в исследование, полное гормональное обследование до РЙТ прошло 37 пациенток, 2 пациентки — только определение АМГ. На повторное полное гормональное обследование явилось 35 пациенток через 3 мес. и 36 пациенток — через 6 мес. При анализе гормональных показателей в динамике, а именно до РЙТ, через 3 и 6 мес. после РЙТ, нами были исключены пациентки, которые не имели полную динамику по каждому из исследуемых параметров.

Для выявления предикторов снижения АМГ как основного и чувствительного гормонального маркера ОР общая группа пациенток сначала была разделена на 2 подгруппы по медиане снижения уровня АМГ (≥50% и <50% через 3 мес., ≥38% и <38% через 6 мес.). В последующем — по уровню АМГ: АМГ≥1,2 нг/мл (соответствует нормальному ОР), АМГ<1,2 нг/мл (соответствует сниженному ОР) через 3 и через 6 мес., при этом из исследования исключены 5 пациенток с исходным уровнем АМГ ниже 1,2 нг/мл.

## Методы

После оперативного лечения диагноз «ДРЩЖ» с определением гистологического типа устанавливался по результатам патологоанатомического исследования в ГНЦ РФ ФГБУ «НМИЦ эндокринологии» Минздрава РФ (далее — НМИЦ эндокринологии). Проводилась послеоперационная стратификация риска рецидива заболевания с выделением трех групп (группа низкого риска, группа промежуточного риска, группа высокого риска) на основании рекомендаций Американской тиреоидологической ассоциации (American Thyroid Association, 2015 г.) для определения дальнейшей тактики ведения — наблюдение или проведение второго этапа лечения — РЙТ [15–17].

Проводился общеклинический осмотр, подробное изучение анамнеза и текущего статуса пациенток с ДРЩЖ перед каждым забором крови для установления соответствия критериям включения и исключения и определения жалоб. Проведен гормональный анализ крови с определением АМГ, ФСГ, ЛГ, E2 (автоматизированная тест-система VITROS 3600). Забор крови проводился утром с 09:00 до 11:00 натощак на 3–5 дни менструального цикла, при невозможности приехать в раннюю фолликулярную фазу пациенткам было предложено приехать в любой день цикла для обследования только на АМГ, так как его уровень стабилен на протяжении всего менструального цикла.

Лабораторные исследования проводили на базе клинико-диагностической лаборатории НМИЦ эндокринологии. Забор крови на исследование проводился 3 раза — после установки окончательного диагноза «ДРЩЖ» в ходе послеоперационного патологоанатомического исследования, но как минимум за месяц до РЙТ; через 3 месяца после РЙТ (на фоне супрессивной терапии); через 6 месяцев после РЙТ (на фоне супрессивной терапии).

Нарушение менструального цикла устанавливалось в соответствии с российскими клиническими рекомендациями «Аномальные маточные кровотечения» 2021 г., «Аменорея и олигоменорея» 2021 г. [18][19]. Сниженный ОР устанавливался при уровне АМГ менее 1,2 нг/мл (Poseidon criteria, клинические рекомендации «Женское бесплодие») [20].

## Статистический анализ

Статистический анализ выполнен с помощью языка программирования Python 3.11 с использованием библиотек scipy 1.11.1 и scikit-learn 1.3.0. Описательная статистика количественных признаков представлена с помощью медиан, первых и третьих квартилей (Me [Q1; Q3]), категориальных признаков — с помощью абсолютных и относительных частот (n (%)). Сравнительный анализ двух независимых групп по количественным признакам выполнен с помощью критерия Манна-Уитни (U-тест), по категориальным признакам — с помощью двустороннего точного критерия Фишера (ТКФ2). Для поиска отрезных точек был выполнен ROC-анализ. Отрезные точки выбирались согласно индексу Юдена. Для отрезных точек были рассчитаны операционные характеристики: диагностическая чувствительность (ДЧ), диагностическая специфичность (ДС), прогностическая ценность положительного результата (ПЦПР) и прогностическая ценность отрицательного результата (ПЦОР) с 95% доверительными интервалами (ДИ).

Уровень статистической значимости был принят равным 0,05. При множественных сравнениях уровень статистической значимости был скорректирован с помощью поправки Бонферрони (Р0).

## Этическая экспертиза

Работа одобрена локальным этическим комитетом при ГНЦ РФ ФГБУ «НМИЦ эндокринологии» Минздрава России. Протокол заседания локального этического комитета №18 от 12.10.2022 г.

## РЕЗУЛЬТАТЫ

Размер выборки составил 39 пациенток с папиллярным раком щитовидной железы, получающих комбинированное лечение по поводу заболевания. Возраст начала терапии 32 года [ 27; 37].

Характеристика пациенток представлена в таблице 1.

**Table:** Таблица 1. Характеристика пациенток с ДРЩЖ

Признак	N	Me [ Q1; Q3] / n (%)
Менархе, годы	39	13 [ 12; 14]
ИМТ, кг/м²	39	23,4 [ 20,5; 26,2]
ИМТ	Дефицит	39	1 (3%)
Норма	22 (56%)
Избыточная масса тела	12 (31%)
Ожирение	4 (10%)
Левотироксин после ТЭ	39	125 [ 125; 150]
ТТГ, мМЕ/л	39	85,66 [ 69,39; 111,56]
ТГ, нг/мл	39	1,43 [ 0,20; 4,12]
АТ-ТГ, МЕ/мл	39	17,76 [ 7,65; 32,48]
Кумулятивная активность, МБк	39	3720 [ 3050; 3838]
Левотироксин после РЙТ, мкг/сут	39	125 [ 125; 150]
ФСГ, Ед/л (исходно)	37	4,8 [ 4,1; 5,7]
ЛГ, Ед/л (исходно)	37	4,10 [ 2,80; 5,40]
АМГ, нг/мл (исходно)	39	4,10 [ 2,34; 5,82]
E2, пмоль/л (исходно)	37	165 [ 130; 220]
ФСГ, Ед/л (через 3 месяца)	35	5,2 [ 3,9; 6,2]
ЛГ, Ед/л (через 3 месяца)	35	3,50 [ 2,74; 5,00]
АМГ, нг/мл (через 3 месяца)	39	2,09 [ 1,05; 3,05]
E2, пмоль/л (через 3 месяца)	37	154 [ 103; 216]
ФСГ, Ед/л (через 6 месяцев)	36	5,2 [ 4,1; 6,1]
ЛГ, Ед/л (через 6 месяцев)	36	3,65 [ 2,60; 4,78]
АМГ, нг/мл (через 6 месяцев)	39	2,31 [ 1,42; 3,37]
E2, пмоль/л (через 6 месяцев)	36	161 [ 121; 211]
НМЦ до постановки диагноза	39	7 (18%)
Беременность в анамнезе до начала терапии	39	19 (49%)
Стадия 1	39	39 (100%)
Стадия по Т	Т1a	39	8 (21%)
T1b	18 (46%)
T2	9 (23%)
T3	4 (10%)
Риск рецидива	Низкий	39	1 (3%)
Промежуточный	27 (69%)
Высокий	11 (28%)
Подготовка к РЙТ	Тироген	39	11 (28%)
Отмена	28 (72%)
НМЦ после РЙТ	39	15 (38%)

После первичного обследования до РЙТ нами была оценена динамика половых гормонов через 3 и 6 месяцев после РЙТ. Результаты представлены в таблице 2.

**Table:** Таблица 2. Сравнительный анализ динамики половых гормонов после РЙТ *Критерий ФридманаПоправка Бонферрони Р0=0,05/4=0,0125

Признак	Исходно (1)	Через 3 месяца (2)	Через 6 месяцев (3)	p*	р, post-hoc
N	Me [ Q1; Q3]	N	Me [ Q1; Q3]	N	Me [ Q1; Q3]
ФСГ, Ед/л	34	4,8 [ 4,1; 5,8]	34	5,2 [ 3,9; 6,2]	34	5,2 [ 4,0; 6,2]	0,905	–
ЛГ, Ед/л	34	4,05 [ 2,42; 5,20]	34	3,45 [ 2,72; 4,88]	34	3,65 [ 2,60; 4,92]	0,262	–
АМГ, нг/мл	39	4,10 [ 2,34; 5,82]	39	2,09 [ 1,05; 3,05]	39	2,31 [ 1,42; 3,37]	<0,001	р1-2<0,001 р1-3<0,001 р2-3<0,001
E2, пмоль/л	36	168 [ 129; 220]	36	150 [ 102; 220]	36	161 [ 121; 211]	<0,001	р1-2=0,010 р1-3=0,123 р2-3=0,031

По результатам нашей работы, только уровень АМГ статистически значимо снижался после РЙТ — медиана снижения АМГ через 3 мес. составила 50% [ 32%; 60%], через 6 мес. — 38% [ 20%; 55%]. Уровень Е2 значимо снижался к 3 мес., а к 6 мес. приближался к исходным значениям.

Сравнительный анализ в зависимости от снижения уровня АМГ через 3 мес. и через 6 мес. представлен в таблицах 3 и 4 соответственно.

**Table:** Таблица 3. Сравнительный анализ пациенток со снижением АМГ через 3 месяца более 50% (N=20) и менее 50% (N=19) Поправка Бонферрони Р0=0,05/20=0,0025¹ U-тест² ТКФ2

Признак	Снижение АМГ ≥ 50%	Снижение АМГ < 50%	p
N	Me [ Q1; Q3] / n (%)	N	Me [ Q1; Q3] / n (%)
Менархе, годы	20	13 [ 12; 14]	19	13 [ 12; 13]	0,714¹
Возраст на момент начала РЙТ, годы	20	36 [ 30; 37]	19	30 [ 26; 34]	0,061¹
ИМТ, кг/м²	20	25,6 [ 20,8; 27,9]	19	21,5 [ 20,4; 25,0]	0,081¹
ИМТ	Дефицит	20	1 (5%)	19	0 (0%)	0,046²
Норма	8 (40%)	14 (74%)
Избыточная масса тела	7 (35%)	5 (26%)
Ожирение	4 (20%)	0 (0%)
Левотироксин после ТЭ	20	125 [ 125; 150]	19	125 [ 112; 150]	0,817¹
ТТГ, мМЕ/л	20	74,45 [ 64,55; 99,93]	19	91,50 [ 75,85; 115,30]	0,177¹
ТГ, нг/мл	20	1,36 [ 0,20; 5,20]	19	1,64 [ 0,21; 2,59]	0,757¹
АТ-ТГ, МЕ/мл	20	16,90 [ 10,59; 56,72]	19	18,09 [ 6,34; 25,51]	0,833¹
Кумулятивная активность, МБк	20	3745 [ 3085; 3925]	19	3720 [ 2990; 3800]	0,694¹
Левотироксин после РЙТ, мкг/сут	20	125 [ 125; 150]	19	125 [ 112; 150]	0,717¹
ФСГ, Ед/л (исходно)	20	4,7 [ 4,2; 5,7]	17	5,0 [ 4,0; 5,7]	0,903¹
ЛГ, Ед/л (исходно)	20	4,05 [ 2,90; 6,30]	17	4,20 [ 2,30; 5,20]	0,647¹
АМГ, нг/мл (исходно)	20	4,26 [ 2,59; 7,22]	19	4,10 [ 1,86; 5,12]	0,191¹
E2, пмоль/л (исходно)	19	179 [ 150; 268]	18	144 [ 118; 192]	0,029¹
НМЦ до постановки диагноза	20	3 (15%)	19	4 (21%)	0,695²
Беременность в анамнезе до терапии	20	11 (55%)	19	8 (42%)	0,527²
Стадия Т	Т1a	20	3 (15%)	19	5 (26%)	0,694²
T1b	11 (55%)	7 (37%)
T2	4 (20%)	5 (26%)
T3	2 (10%)	2 (11%)
Риск рецидива	Низкий	20	0 (0%)	19	1 (5%)	0,218²
Промежуточный	16 (80%)	11 (58%)
Высокий	4 (20%)	7 (37%)
Подготовка к РЙТ	Тироген	20	3 (15%)	19	8 (42%)	0,082²
Отмена	17 (85%)	11 (58%)
НМЦ после РЙТ	20	7 (35%)	19	8 (42%)	0,748²

**Table:** Таблица 4. Сравнительный анализ пациенток со снижением АМГ через 6 месяцев более 38% (N=20) и менее 38% (N=19) Поправка Бонферрони Р0=0,05/20=0,0025¹ U-тест² ТКФ2

Признак	Снижение АМГ ≥ 38%	Снижение АМГ < 38%	p
N	Me [ Q1; Q3] / n (%)	N	Me [ Q1; Q3] / n (%)
Менархе, годы	20	13 [ 12; 14]	19	13 [ 12; 13]	0,883¹
Возраст на момент начала терапии, годы	20	34 [ 30; 37]	19	30 [ 26; 36]	0,083¹
ИМТ, кг/м²	20	25,6 [ 21,3; 27,0]	19	20,9 [ 20,2; 25,0]	0,140¹
ИМТ	Дефицит	20	1 (5%)	19	0 (0%)	0,107²
Норма	8 (40%)	14 (74%)
Избыточная масса тела	9 (45%)	3 (16%)
Ожирение	2 (10%)	2 (11%)
Левотироксин после ТЭ	20	125 [ 125; 150]	19	125 [ 112; 150]	0,487¹
ТТГ, мМЕ/л	20	74,45 [ 61,62; 102,50]	19	91,50 [ 75,85; 115,30]	0,169¹
ТГ, нг/мл	20	2,29 [ 0,35; 5,20]	19	1,24 [ 0,20; 1,86]	0,211¹
АТ-ТГ, МЕ/мл	20	16,90 [ 10,59; 56,72]	19	17,85 [ 6,34; 25,51]	0,593¹
Кумулятивная активность, МБк	20	3745 [ 3075; 3829]	19	3720 [ 3050; 3855]	0,966¹
Левотироксин после РЙТ, мкг/сут	20	125 [ 125; 150]	19	125 [ 125; 150]	0,601¹
ФСГ, Ед/л (исходно)	20	4,9 [ 4,2; 5,7]	17	4,7 [ 4,0; 5,6]	0,532¹
ЛГ, Ед/л (исходно)	20	3,90 [ 2,27; 5,02]	17	4,50 [ 2,90; 5,40]	0,512¹
АМГ, нг/мл (исходно)	20	4,90 [ 2,94; 7,22]	19	3,15 [ 2,12; 5,04]	0,084¹
E2, пмоль/л (исходно)	19	175 [ 144; 256]	18	156 [ 117; 210]	0,098¹
НМЦ до постановки диагноза	20	4 (20%)	19	3 (16%)	1,000²
Беременность в анамнезе до терапии	20	11 (55%)	19	8 (42%)	0,527²
Стадия Т	Т1a	20	3 (15%)	19	5 (26%)	0,694²
T1b	11 (55%)	7 (37%)
T2	4 (20%)	5 (26%)
T3	2 (10%)	2 (11%)
Риск рецидива	Низкий	20	1 (5%)	19	0 (0%)	0,384²
Промежуточный	15 (75%)	12 (63%)
Высокий	4 (20%)	7 (37%)
Подготовка к РЙТ	Тироген	20	5 (25%)	19	6 (32%)	0,731²
Отмена	15 (75%)	13 (68%)
НМЦ после РЙТ	20	8 (40%)	19	7 (37%)	1,000²

Статистически значимые различия между подгруппами не обнаружены, так как степень снижения уровня АМГ не зависела ни от одного из изучаемых параметров. Сравнительный анализ подгрупп по уровню АМГ через 3 месяца представлен в таблице 5.

**Table:** Таблица 5. Сравнительный анализ групп пациенток с уровнем АМГ в референсе (N=24) и ниже референса (N=10) через 3 месяца Поправка Бонферрони Р0=0,05/20=0,0025¹ U-тест² ТКФ2

Признак	АМГ ≥ 1,2 нг/мл	АМГ < 1,2 нг/мл	p
N	Me [ Q1; Q3] / n (%)	N	Me [ Q1; Q3] / n (%)
Менархе, годы	24	13 [ 12; 14]	10	13 [ 12; 13]	0,752¹
Возраст на момент начала терапии, годы	24	28 [ 26; 32]	10	37 [ 34; 38]	0,001¹
ИМТ, кг/м²	24	22,9 [ 20,6; 25,9]	10	24,4 [ 20,5; 27,0]	0,326¹
ИМТ	Дефицит	24	0 (0%)	10	1 (10%)	0,145²
Норма	15 (62%)	3 (30%)
Избыточная масса тела	7 (29%)	4 (40%)
Ожирение	2 (8%)	2 (20%)
Левотироксин после ТЭ	24	125 [ 125; 150]	10	131 [ 106; 150]	0,861¹
ТТГ, мМЕ/л	24	87,82 [ 70,35; 114,02]	10	91,07 [ 71,35; 99,98]	0,970¹
ТГ, нг/мл	24	1,53 [ 0,30; 4,14]	10	0,32 [ 0,07; 3,78]	0,298¹
АТ-ТГ, МЕ/мл	24	17,54 [ 2,17; 28,31]	10	17,80 [ 14,96; 72,90]	0,650¹
Кумулятивная активность, МБк	24	3745 [ 3045; 3800]	10	3823 [ 3190; 3962]	0,496¹
Левотироксин после РЙТ, мкг/сут	24	125 [ 125; 150]	10	131 [ 125; 150]	0,953¹
ФСГ, Ед/л (исходно)	22	5,0 [ 4,1; 5,7]	10	4,7 [ 3,8; 6,2]	0,968¹
ЛГ, Ед/л (исходно)	22	4,45 [ 2,82; 6,28]	10	3,50 [ 2,23; 4,95]	0,339¹
АМГ, нг/мл (исходно)	24	5,12 [ 4,06; 6,14]	10	2,58 [ 1,88; 3,56]	0,010¹
E2, пмоль/л (исходно)	22	159 [ 132; 243]	10	166 [ 130; 216]	0,968¹
НМЦ до постановки диагноза	24	3 (12%)	10	2 (20%)	0,145²
Беременность в анамнезе до терапии	24	7 (29%)	10	9 (90%)	0,002²
Стадия Т	Т1a	24	4 (17%)	10	2 (20%)	0,948²
T1b	10 (42%)	5 (50%)
T2	7 (29%)	2 (20%)
T3	3 (12%)	1 (10%)
Риск рецидива	Низкий	24	1 (4%)	10	0 (0%)	0,205²
Промежуточный	14 (58%)	9 (90%)
Высокий	9 (38%)	1 (10%)
Подготовка к РЙТ	Тироген	24	5 (21%)	10	4 (40%)	0,395²
Отмена	19 (79%)	6 (60%)
НМЦ после РЙТ	24	9 (38%)	10	1 (10%)	0,215²

Статистически значимые различия обнаружены по возрасту на момент начала РЙТ (Р=0,001) и наличию беременности в анамнезе (Р=0,002). На уровне статистической тенденции были обнаружены различия по исходному уровню АМГ (Р=0,01). Был проведен ROC-анализ для данных количественных показателей с целью оценки их прогностических способностей. ROC-анализ возраста представлен на рисунке 2.

**Figure:**
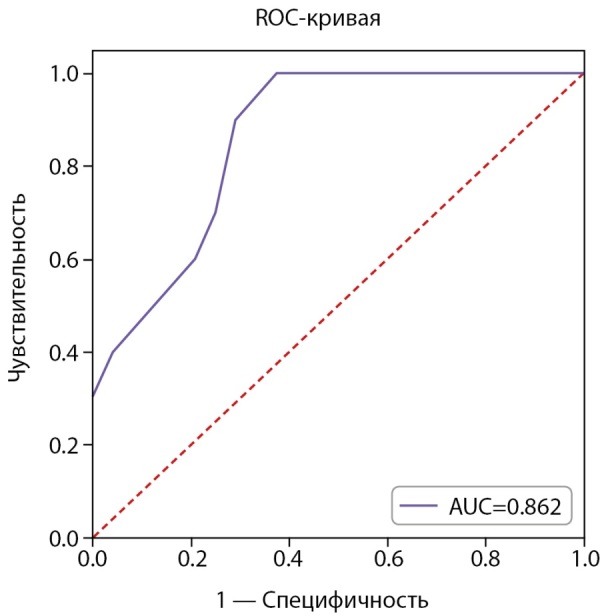
Рисунок 2. ROC-анализ возраста пациенток на начало РЙТ для прогнозирования снижения АМГ ниже 1,2 нг/мл через 3 мес. (N=34).

AUC=0,862 (95% ДИ: 0,707–1,000), что свидетельствует о средней прогностической способности возраста. Отрезная точка, согласно индексу Юдена, была выбрана равной 31 году. Матрица классификации пациенток, согласно отрезной точке, представлена в таблице 6.

**Table:** Таблица 6. Матрица классификации пациенток старше (N=19) и младше (N=15) 31 года в зависимости от уровня АМГ через 3 месяца

	Возраст на момент начала терапии
≥ 31 года	< 31 года
АМГ через 3 месяца	< 1,2 нг/мл	10	0
≥ 1,2 нг/мл	9	15

Операционные характеристики точки: ДЧ=53% (95% ДИ: 37%–53%), ДС=100% (95% ДИ: 80%–100%), ПЦПР=100% (95% ДИ: 71%–100%), ПЦОР=63% (95% ДИ: 50%–63%). Таким образом, у пациенток 31 года и старше вероятность снижения АМГ менее 1,2 нг/мл через 3 месяца 71%–100%.

ROC-анализ исходного уровня АМГ представлен на рисунке 3.

**Figure:**
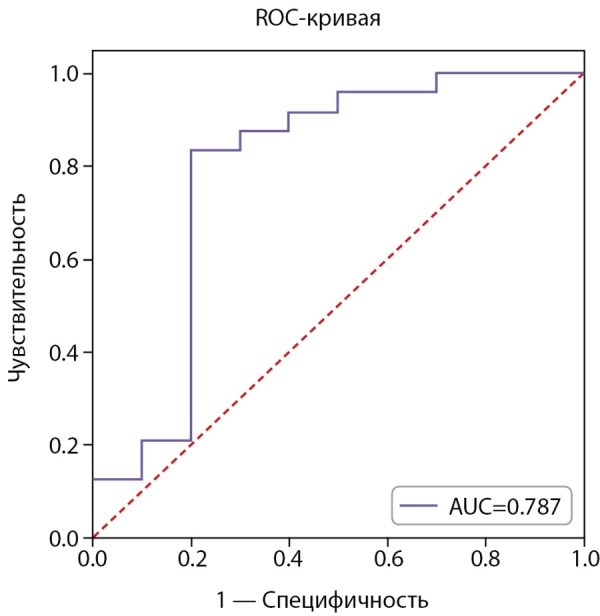
Рисунок 3. ROC-анализ исходного уровня АМГ для прогнозирования снижения АМГ ниже 1,2 нг/мл через 3 мес. (N=34).

AUC=0,787 (95% ДИ: 0,632–0,943), что свидетельствует о средней прогностической способности исходного уровня АМГ. Отрезная точка, согласно индексу Юдена, была выбрана равной 3,93 нг/мл. Матрица классификации пациенток, согласно отрезной точке, представлена в таблице 7.

**Table:** Таблица 7. Матрица классификации пациенток с исходным уровнем АМГ менее (N=12) и более (N=22) 3,93 нг/мл в зависимости от уровня АМГ через 3 месяца

	АМГ (исходно)
< 3,93 нг/мл	≥ 3,93 нг/мл
АМГ через 3 месяца	< 1,2 нг/мл	8	2
≥ 1,2 нг/мл	4	20

Операционные характеристики точки: ДЧ=67% (95% ДИ: 41%–80%), ДС=91% (95% ДИ: 77%–98%), ПЦПР=80% (95% ДИ: 50%–96%), ПЦОР=83% (95% ДИ: 71%–90%). Таким образом, у пациенток с исходным уровнем АМГ ниже 3,93 нг/мл вероятность снижения АМГ менее 1,2 нг/мл через 3 месяца 50%–96%.

Сравнительный анализ групп с уровнем АМГ в референсе и ниже референса через 6 месяцев выполнить невозможно в связи с тем, что размер выборки пациенток с уровнем АМГ <1,2 нг/мл через 6 месяцев составляет 2 человека.

## ОБСУЖДЕНИЕ

Первый этап лечения ДРЩЖ — тиреоидэктомия различного объема, и при необходимости вмешательство на лимфатических узлах не является гонадотоксичным методом лечения при условии компенсации послеоперационного гипотиреоза [21]. Однако следующий этап лечения, радионуклидная терапия ¹³¹I, а также подготовка к нему и к радионуклидной диагностике в процессе дальнейшего динамического наблюдения пациента могут влиять на функциональное состояние яичников и репродуктивную функцию.

К настоящему времени описаны различные вторичные осложнения РЙТ, возникающие в первые сутки после терапии или развивающиеся отсрочено [22][23]. Одной из основных причин нежелательных явлений и осложнений становится действие ионизирующего излучения не только в области остаточной тиреоидной ткани и накапливающих ¹³¹I очагах опухоли, но и за их пределами.

При этом экспериментально доказано, что яичники не обладают способностью захватывать и накапливать ¹³¹I, но получают дозу облучения из притекающей к ним крови, от мочевого пузыря и толстого кишечника, участвующих в выведении ¹³¹I, а также от метастазов ДРЩЖ, находящихся в малом тазу и накапливающих ¹³¹I [24]. Потенцирование процессов оксидативного стресса при РЙТ способствует повреждению ДНК, что может отражаться на пуле растущих фолликулов и влиять на стероидогенез [25].

Изначально большинство исследований, посвященных функциональному состоянию яичников и фертильности женщин с ДРЩЖ, прошедших оперативное и радионуклидное лечение, основывались на оценке наступления беременности, исследовании менструального цикла и регистрации климактерических симптомов, а также на определении уровня ФСГ, что на самом деле не позволяет судить об ОР [26–28]. Появление АМГ в качестве диагностического маркера количества растущих фолликулов позволило точнее оценивать влияние комбинированного лечения на женскую репродуктивную систему.

Уровень АМГ в сыворотке крови наравне с количеством антральных фолликулов (КАФ) — наиболее часто применяемые и точные маркеры ОР, которые позволяют спрогнозировать ответ яичников на один из первых этапов программ ВРТ — овариальную стимуляцию — и персонализированно подобрать план лечения [29].

Впервые оценка риска гонадотоксичности РЙТ у пациенток с ДРЩЖ с помощью исследования АМГ была проведена в 2016 г., Аcibucu и соавт. отметили более низкие средние значения АМГ у женщин с ДРЩЖ, получивших РЙТ, по сравнению со здоровыми женщинами того же возраста (Р<0,038) [30]. В ходе проспективных работ в этой области выявили, что минимальный уровень АМГ наблюдается через 3 месяца после РЙТ с лишь частичным его восстановлением к концу первого года после лечения [31][32]. При этом, несмотря на значимое изменение АМГ, уровни ФСГ, ЛГ и Е2 практически не отличались от таковых до терапии (P>0,05) [32]. Основными предикторами более значимого снижения АМГ стали возраст старше 35 лет и количество курсов РЙТ [33]. Кроме АМГ, Adamska и соавт. использовали ингибин В, КАФ и ФСГ для оценки ОР у пациенток с ДРЩЖ. Статистически значимое снижение КАФ (P=0,03), АМГ (P<0,01) и ингибина B (P=0,03) установлено через 1 год после РЙТ по сравнению с исходными значениями, а вот уровень ФСГ оставался прежним [34].

В настоящей работе было рассмотрено влияние комбинированного лечения ДРЩЖ на уровни ФСГ, ЛГ, Е2 и АМГ у 39 пациенток репродуктивного возраста без установленного ранее диагноза бесплодия и других гинекологических заболеваний и их лечения, которые потенциально могли бы отразиться на результатах гормонального обследования и привести к ложным результатам.

Наблюдаемые нами изменения соотносятся с результатами предыдущих работ в этой области — уровень АМГ статистически значимо снижается после РЙТ и остается ниже исходных значений в течение первых 6 мес. (Р<0,001) на фоне получаемой пациентами супрессивной терапии. В отличие от предыдущих работ, нами было установлено значимое снижение уровня Е2 через 3 мес. после терапии с его дальнейшим повышением практически до исходных значений. Эти наблюдения могут быть обусловлены нарушением фолликулогенеза и интенсификацией процессов атрезии фолликулов, особенно в первые 3 мес. после РЙТ, когда отмечается надир АМГ.

При этом важно отметить, что хоть уровень АМГ и отражает количество растущих фолликулов, его применение для предикции качества ооцитов все еще требует углубленного изучения [35][36]. В будущем возможно использование АМГ, а именно его крайне низкого или неопределяемого уровня, как прогностического маркера раннего наступления менопаузы или преждевременной недостаточности яичников. Однако данные, полученные в популяционных исследованиях, нельзя экстраполировать на конкретного пациента [37][38]. В настоящее время главным фактором, определяющим уровень АМГ, остается возраст. АМГ отражает пул растущих фолликулов, он продуцируется гранулезными клетками преантральных и малых антральных фолликулов и ингибирует переход фолликулов из примордиального пула в антральный, при этом его уровень может коррелировать с количеством примордиальных фолликулов на фоне комбинированной терапии ДРЩЖ [39][40].

Мы предполагаем, что снижение АМГ к 3 месяцу от РЙТ может быть связано с запуском процессов апоптоза примордиальных фолликулов и атрезии растущих фолликулов, а последующее повышение АМГ к 6 месяцу — с временным повышением числа преантральных и малых антральных фолликулов после вовлечения примордиальных фолликулов в растущий пул. Другими словами, мы можем наблюдать активацию резерва фолликулов у части пациентов, однако для того, чтобы сделать выводы о долгосрочном влиянии данных изменений, требуется более длительный срок наблюдения, чем в настоящем исследовании, как минимум 12–24 мес. Подобное длительное наблюдение было проведено van Velsen EFS и соавт. Ими было установлено, что уровень АМГ снижался в среднем на 55% в течение первых 12 мес. после 1 курса РЙТ и выходил на плато в последующем. Однако у пациенток, прошедших более 1 курса РЙТ, наблюдалось дальнейшее снижение АМГ — на 85% через 48 месяцев. Возможно, в случае пациенток, у которых отмечается значимое снижение уровня АМГ без его дальнейшего выхода на плато, мишенью ¹³¹I являются и ооциты, и гранулезные клетки, а патофизиологические аспекты снижения ОР связаны с запуском процессов апоптоза, как это происходит в случае использования алкилирующих агентов, только при РЙТ это связано с радиобиологическими свойствами ¹³¹I. Однако для того, чтобы судить о РЙТ-индуцированных процессах в яичниках, исходя из молекулярных механизмов, и, в частности, делать выводы о потере примордиального пула, требуется проведение гистологического исследования ткани яичников [10].

Что касается рекомендаций по планированию беременности, то зарубежные и российские руководства декларируют необходимость воздержания от зачатия в течение первых 6–12 мес. после РЙТ [15][16]. Несмотря на это, в общественной практике врачи зачастую могут рекомендовать избегать зачатия куда более продолжительный срок, в связи с чем пациентки откладывают беременность на поздний репродуктивный возраст, когда шансы на успешное зачатие и без учета проводимой терапии могут быть снижены. Кроме того, РЙТ сопряжена с определенным стрессом, развитием депрессии и повышенной тревожностью, что может способствовать гормональным нарушениям, усугубить страх перед планированием беременности и отложить зачатие [41].

Учитывая эти особенности, необходимо проводить беседу с пациентками репродуктивного возраста до начала второго этапа лечения, привлекая специалистов смежных областей, для своевременного выявления нарушения репродуктивной функции и предикторов ее изменения на фоне проводимого лечения, а также оценки риска других потенциальных осложнений РЙТ.

## Клиническая значимость результатов

Клиническая значимость определения АМГ у пациенток с ДРЩЖ продиктована несколькими основными моментами. Первое — высокой вероятностью снижения АМГ в процессе лечения в результате непосредственного влияния радиоактивного йода на фолликулогенез, а также неоднократной смены тиреоидного статуса. Второе — необходимостью своевременного использования мер по сохранению фертильности, особенно в том случае, если уже до РЙТ наблюдается сниженный ОР по данным АМГ и/или КАФ, а также если прогнозируется снижение ОР в ходе лечения, а пациентка планирует беременность в будущем.

С нашей точки зрения, учитывая ожидаемое снижение АМГ после РЙТ по поводу ДРЩЖ, исходно низкий уровень АМГ и, опираясь на полученные результаты настоящей работы, возраст женщины старше 31 года должны быть сигналом для направления пациентки к специалистам репродуктивной медицины с целью обсуждения использования мер по сохранению фертильности в каждом конкретном случае. Однако важно отметить, что АМГ не отражает качество ооцитов, и даже при сохранном ОР мы не можем утверждать, что терапия или другие факторы не повлияли на способность яичников давать здоровую яйцеклетку.

## Ограничения исследования

Основными ограничениями исследования являются небольшая выборка пациенток с ДРЩЖ и непродолжительный период наблюдения. Мы убеждены, что требуется как минимум в течение первого года отслеживать АМГ для того, чтобы провести более точную оценку наблюдаемых изменений. Кроме того, дополнительной пользой для оценки ОР в данной когорте пациенток будет проведение фолликулометрии и подсчет КАФ совместно с определением АМГ на 3 день менструального цикла как минимум до РЙТ и спустя 6 мес. после лечения.

## ЗАКЛЮЧЕНИЕ

Комбинированное лечение ДРЩЖ приводит к снижению ОР по данным АМГ у большинства женщин детородного возраста, что потенциально может сказаться на фертильности и продолжительности репродуктивного периода. Мы рекомендуем оценивать уровень АМГ у пациенток детородного возраста с ДРЩЖ, которым планируется послеоперационная РЙТ, по возможности совмещая лабораторную диагностику с подсчетом КАФ с помощью ультразвукового исследования для исключения дискордантности показателей, отражающих ОР. Оценка ОР может быть полезным инструментом для прогнозирования изменений в яичниках в процессе лечения и позволит своевременно направить женщину к акушеру-гинекологу и/или специалисту репродуктивной медицины для решения вопроса о дообследовании и применении мер по сохранению фертильности.

## ДОПОЛНИТЕЛЬНАЯ ИНФОРМАЦИЯ

Источник финансирования. Данная работа выполнена в соответствии с планом государственного задания. Регистрационный номер 123021000041-6.

Конфликт интересов. Авторы декларируют отсутствие явных и потенциальных конфликтов интересов, связанных с публикацией настоящей статьи.

Участие авторов. Все авторы внесли значимый вклад в проведение исследования и подготовку статьи, прочли и одобрили финальную версию статьи перед публикацией.
